# Castleman’s Disease with Pulmonary Artery Aneurysm: A Rare Presentation of Behçet’s Disease

**DOI:** 10.7759/cureus.7647

**Published:** 2020-04-12

**Authors:** Mais Al-Sardi, Deemah Abdulhadi, Faiza Al-Jishi, Khaled Deraan

**Affiliations:** 1 Internal Medicine, King Fahad Specialist Hospital, Dammam, SAU; 2 Rheumatology, King Fahad Specialist Hospital, Dammam, SAU

**Keywords:** castleman’s disease, pulmonary artery aneurysm, behcet’s disease

## Abstract

Diagnosing Behcet’s disease (BD) in a patient already diagnosed with Castelman’s disease (CD) is rare. There are only a few cases reported in the literature and all of them were diagnosed as BD prior to the patient experiencing symptoms and signs of CD. We present a patient who was initially diagnosed as having CD. However, after being managed with chemotherapy, specifically after the fourth cycle, the patient was found to have an incidental finding of pulmonary artery aneurysm, which led to the diagnosis of BD. For that, he received the appropriate management of high-dose steroid, azathioprine, and oral anticoagulant. Currently, the patient is doing well, and the latest computed tomography scan showed complete resolution of his pulmonary aneurysm. We suggest taking a thorough history from all patients with BD symptoms and signs, especially in CD patients as they may overlap, for early diagnosis and to prevent complications.

## Introduction

The presence of Behçet’s disease (BD) with Castleman’s disease (CD) is quite rare, as only a few cases have been reported worldwide, all of which were diagnosed as BD prior to the patient experiencing manifestations of CD. The patient in our case was initially diagnosed as CD and found to have an incidental finding of pulmonary aneurysm, which led to the diagnosis of BD.

## Case presentation

**Figure 1:**
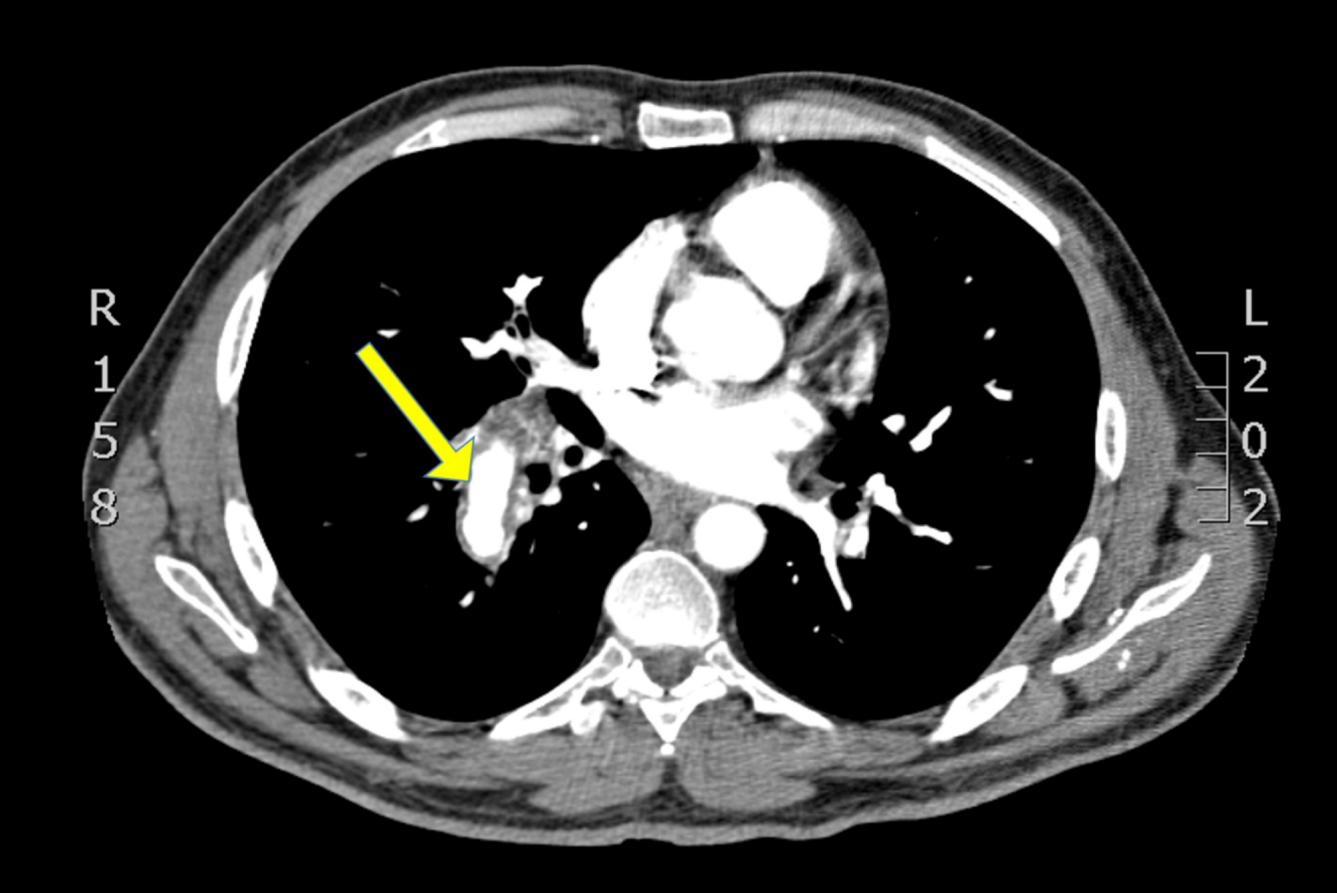
Chest CT after fourth cycle of chemotherapy showing dilated right pulmonary artery with wall thickening CT, computed tomography

Our patient is a 46-year-old Saudi man who was referred to our tertiary care center with B-symptoms (fever and significant unintentional weight loss), recurrent pneumonia, and lymphadenopathy. His computed tomography (CT) scan showed hilar, mediastinal, para-aortic, and inguinal lymphadenopathy with enlarged liver and spleen. Inguinal lymph node biopsy confirmed CD hyaline vascular type. He was treated with six cycles of R-CHOP (rituximab, cyclophosphamide, doxorubicin, vincristine, prednisone) chemotherapy, which he tolerated very well with minor expected side effects. After the fourth cycle of chemotherapy, he underwent a regular follow-up CT scan of the chest, abdomen, and pelvis. It showed dilation of the posterior portion of both main pulmonary arteries more prominent on the right with wall thickening. There was also a partial filling defect and recanalization, suggesting a chronic pulmonary artery aneurysm as this may have developed before starting his chemotherapy ( Figure 1).

It also showed an enlargement of the hilar and subcarinal lymph nodes. Both findings were new compared to old images. 

Two weeks after the fifth cycle, the patient developed hemoptysis and reported concerns of gritty eyes. Upon further assessment, he had recurrent bouts of painful orogenital ulcers and recurrent painful lesions on his legs, which were later diagnosed as erythema nodosum. He also had acneiform lesions involving his face and trunk, painful red eyes, and recurrent knee arthritis with effusion along with his previous constitutional symptoms.

Laboratory investigations showed a normal cell count and that his inflammatory markers were not elevated (C-reactive protein and erythrocyte sedimentation rate). His autoimmune profile was negative (antinuclear antibodies, anti-DNA, anti-Sjögren’ s-syndrome-related antigen A, anti-cyclic citrullinated peptides, anticardiolipin antibodies, lupus anticoagulant), and his human leukocyte antigen-B51 was negative. His echocardiogram showed a normal study. 

**Figure 2:**
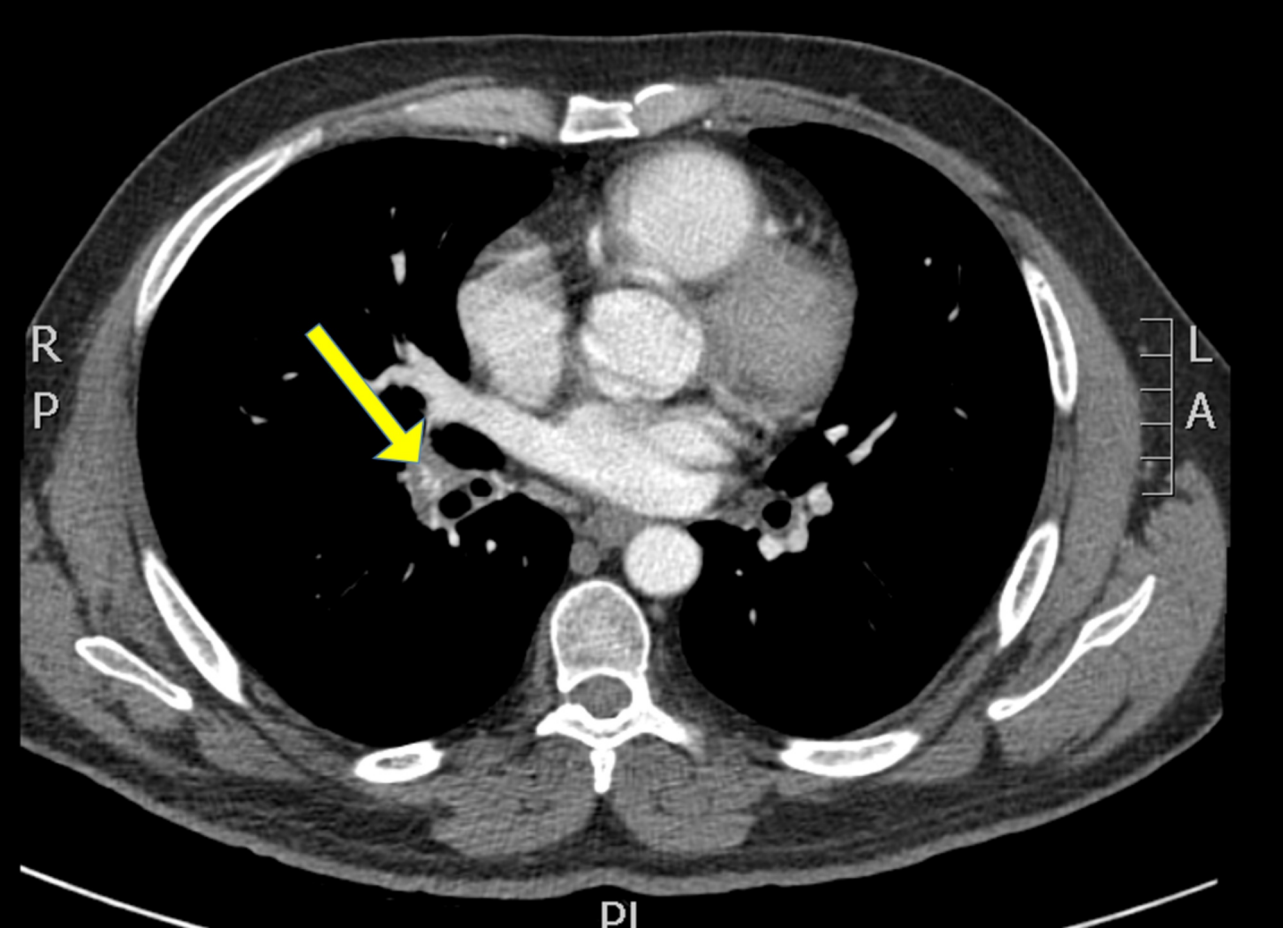
Chest CT showing significant decrease in the pulmonary artery size CT, computed tomography

Based on his presentation, the patient was diagnosed as having BD by fulfilling the International Criteria for Behcet’s disease (ICBD) and the International Study Group (ISG) with a pulmonary aneurysm. Accordingly, he was hospitalized and started on hydrocortisone 100 mg intravenously every six hours, azathioprine 50 mg orally daily, and enoxaparin 80 mg twice daily subcutaneously based on his weight. As a result, his symptoms started to improve until they completely resolved. A repeated chest CT scan (done five months after the previous scan) showed marked improvement of his pulmonary aneurysm (Figure 2).

Currently, he is following up with the rheumatology outpatient department and doing well, with no recurrence of ulcers and no signs of active disease. He is being maintained on prednisone 2.5 mg orally daily, azathioprine 150 mg orally daily, colchicine 500 mcg orally daily, and rivaroxaban 20 mg orally daily.

## Discussion

CD is a lymphoproliferative disorder, usually presenting with lymphadenopathy and multiple organ dysfunction [1]. The presence of BD with CD is quite rare, as only a few cases have been reported worldwide. Reported cases include young patients who were diagnosed with BD prior to the diagnosis of CD and one case associated with the relapsed phase of the disease years after receiving the treatment of CD [2-3]. None of the reported cases had a similar finding of the incidental chronic pulmonary aneurysm as a leading sign to diagnose BD, as only 25% to 30% of patients with BD will have vascular involvement, 10% to 15% of which account for arterial involvement [4]. 

In our case, the patient was middle-aged and had symptoms that were suggestive of BD years before receiving a diagnosis of CD, although he never sought medical advice for the same. Even after he was diagnosed as having CD and a genital ulcer was detected, it was thought to be related to his primary disease and not due to another diagnosis. However, when a chronic pulmonary aneurysm was incidentally found on his follow-up CT scan after receiving four cycles of chemotherapy and reviewing the symptoms he experienced and the signs found on examination, he was diagnosed with BD. The diagnosis was made by applying ICBD criteria, and he was assigned seven points due to manifestations of genital aphthosis, ocular lesion, oral aphthosis, and skin and vascular lesions. Our patient also fit the ISG criteria, as he had recurrent oral ulcers (more than three episodes in one year) in addition to recurrent genital ulcers, skin manifestations, and uveitis [5].

Patients with BD would present most commonly with oro-genital ulcers and arthritis along with pulmonary aneurysm. However, the presence of a pulmonary aneurysm carries a poor prognosis [4]. In such cases, CT scan is the radiological investigation of choice for diagnosis [6]. 

Our patient, fortunately, received proper management (prednisolone, cyclophosphamide, and azathioprine), and his symptoms, as mentioned above, improved. He showed a marked improvement of the aneurysmal dilation on CT scan (Figure 1), and his response to treatment was similar to what is reported in the literature [7-8]. 

## Conclusions

In all CD patients who present with BD symptoms and signs such as gritty eyes and erythema nodosum, as seen in our case, a high index of suspicion is required along with a thorough clinical assessment to diagnose BD early, as overlap of BD with CD is quite rare. This can lead to disease control and resolved symptoms and complications including pulmonary aneurysms.
